# Discovery of the world’s highest-dwelling mammal

**DOI:** 10.1073/pnas.2005265117

**Published:** 2020-07-16

**Authors:** Jay F. Storz, Marcial Quiroga-Carmona, Juan C. Opazo, Thomas Bowen, Matthew Farson, Scott J. Steppan, Guillermo D’Elía

**Affiliations:** ^a^School of Biological Sciences, University of Nebraska, Lincoln, NE 68588;; ^b^Instituto de Ciencias Ambientales y Evolutivas, Facultad de Ciencias, Universidad Austral de Chile, Valdivia, Chile, 5090000;; ^c^Millennium Nucleus of Ion Channels-Associated Diseases, Santiago, Chile, 8380453;; ^d^Department of Anthropology, California State University, Fresno, CA 93740;; ^e^Modoc Medical Center, Alturas, CA 96101;; ^f^Department of Biological Science, Florida State University, Tallahassee, FL 32306

**Keywords:** high altitude, Andes, range limits, hypoxia, *Phyllotis*

## Abstract

Environmental limits of animal life are invariably revised when the animals themselves are investigated in their natural habitats. Here we report results of a scientific mountaineering expedition to survey the high-altitude rodent fauna of Volcán Llullaillaco in the Puna de Atacama of northern Chile, an effort motivated by video documentation of mice (genus *Phyllotis*) at a record altitude of 6,205 m. Among numerous trapping records at altitudes of >5,000 m, we captured a specimen of the yellow-rumped leaf-eared mouse (*Phyllotis xanthopygus rupestris*) on the very summit of Llullaillaco at 6,739 m. This summit specimen represents an altitudinal world record for mammals, far surpassing all specimen-based records from the Himalayas and other mountain ranges. This discovery suggests that we may have generally underestimated the altitudinal range limits and physiological tolerances of small mammals simply because the world’s high summits remain relatively unexplored by biologists.

The environmental limits of animal life have always fascinated biologists, and new discoveries about organismal adaptability continually force us to revise our assumptions about such limits. At high altitude, endothermic vertebrates are forced to cope with a combination of environmental stressors, the most salient of which are the reduced partial pressure of oxygen (hypoxia) and freezing temperatures. Nonetheless, numerous alpine mammals and birds have evolved physiological capacities for meeting such challenges (1–3) and are capable of surviving at surprisingly lofty altitudes so long as food is available.

Upper altitudinal limits of wild mammals are generally thought to fall in the range 5,200 m to 5,800 m above sea level (4–6). Such limits are surely dictated by food availability in addition to physiological capacities for tolerating hypoxia and extreme cold. The altitudinal range limits of alpine birds and mammals are often not known with certainty, due to scanty survey data in inaccessible highland regions, and many published records in the scientific literature are surpassed by sightings reported by members of mountaineering expeditions.

Motivated by reported sightings of mice living at record altitudes, we organized a scientific mountaineering expedition to survey the rodent fauna of Volcán Llullaillaco and the surrounding altiplano/Puna de Atacama of northern Chile. Llullaillaco (6,739 m) is the second-highest active volcano in the world and straddles the border between Chile and Argentina. Our trapping results challenge current thinking about physiological and ecological constraints on the altitudinal range limits of mammals and indicate that the world’s highest summits are not as barren as once believed.

## Results and Discussion

**Fig. 1.**
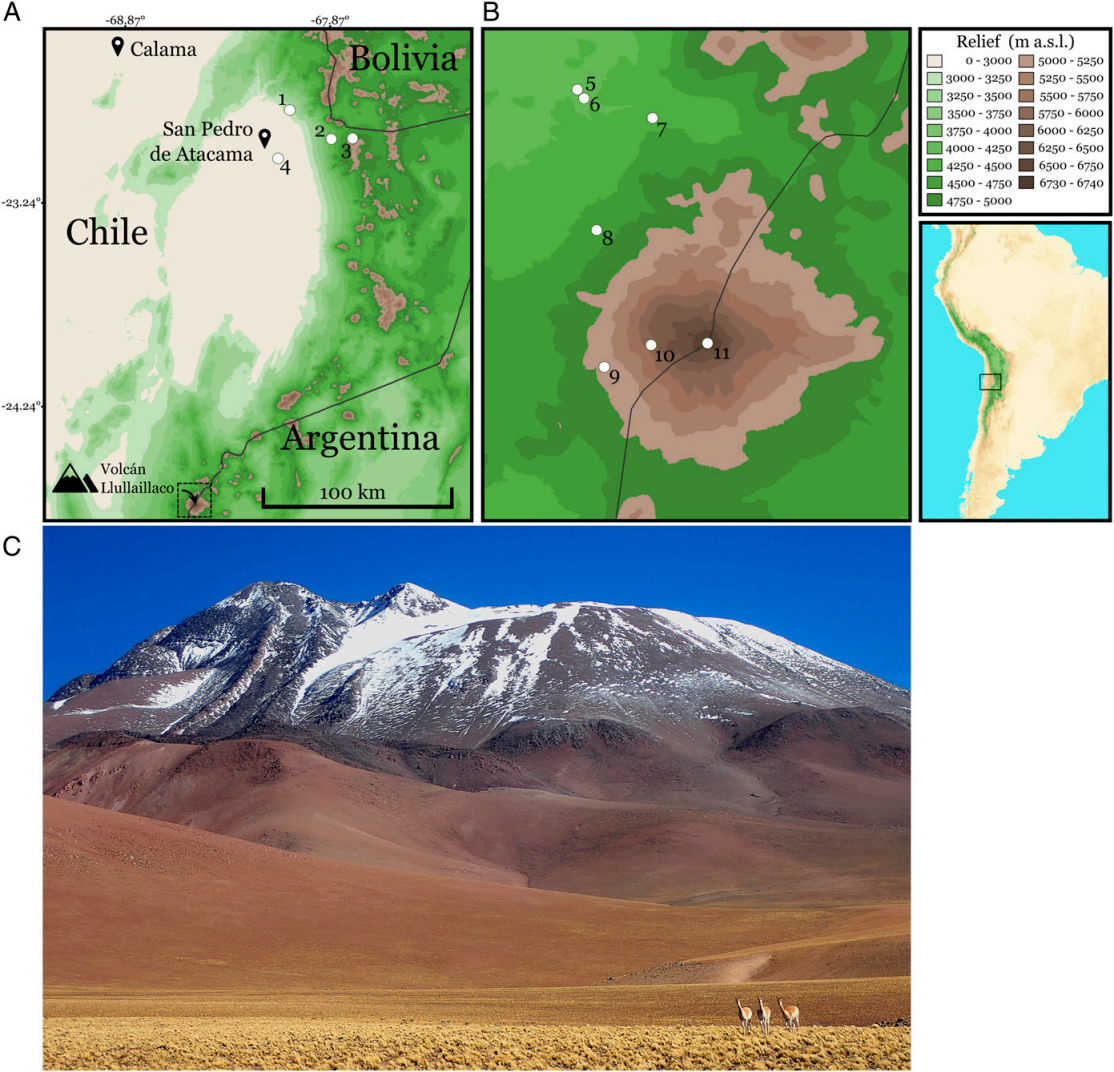
High-altitude survey. Map showing collecting localities in the altiplano and Puna de Atacama (*A*), including Volcán Llullaillaco (*B*), Región de Antofagasta, Chile. (*C*) View of Volcán Llullaillaco (6,739 m [24°43.235′S, 68°32.208′W]) from the west.

On a 2013 mountaineering expedition to Volcán Llullaillaco, M.F. and T.B. filmed a mouse (identified as *Phyllotis* spp.) scurrying across a snowfield at 6,205 m above sea level (24°43.052′S, 68°33.323′W) (Movie S1), an altitude that exceeds existing records for wild mammals. This sighting motivated a subsequent high-altitude trapping expedition in February 2020, led by J.F.S., M.Q.-C., and G.D. During this expedition, we live-trapped rodents from ecologically diverse sites on the altiplano and puna spanning >4,300 m of vertical relief (Fig. 1). On Volcán Llullaillaco, we live-trapped rodents in and around Aguadas de Zorritas (4,140 m to 4,360 m), base camp at Ruta Normal (4,620 m), base camp at Ruta Sur (5,070 m), high camp at Ruta Sur (5,850 m), and the volcano summit (6,739 m). In total, we collected museum voucher specimens of 80 mice representing four species: Andean altiplano mouse (*Abrothrix andina*), altiplano laucha (*Eligmodontia puerulus*), yellow-rumped leaf-eared mouse (*Phyllotis xanthopygus*), and Lima leaf-eared mouse (*Phyllotis limatus*). We collected *Eligmodontia puerulus* and *Abrothrix andina* at maximum altitudes of 4,099 and 4,620 m, respectively; these altitudes approximate or exceed previous records for these species (7, 8). Our altitudinal records for *P. limatus* and *P. xanthopygus* (5,070 and 6,739 m, respectively) far exceed existing records for both species (9–11).

We captured the 6,739-m specimen of *P. xanthopygus* on the very summit of Llullaillaco (24°43.235′S, 68°32.208′W) (Movie S2). This summit specimen represents an altitudinal world record for mammals, far surpassing all specimen-based records from the Himalayas and elsewhere in the Andes. An extensive review of published accounts indicates that the large-eared pika, *Ochotona macrotis* (Lagomorpha), was the previous record holder. Although the highest specimen-based records for this species are from 5,182 m in the Himalayas (US National Museum 198648 and 198649), credible sightings at 6,130 m were reported from a 1921 Everest expedition (12).

**Fig. 2.**
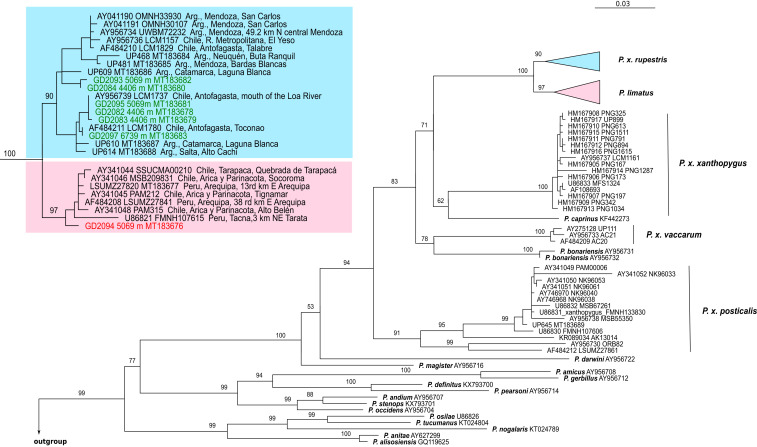
Phylogeny showing the placement of haplotypes of high-altitude *Phyllotis* specimens from Volcán Llullaillaco. Maximum likelihood tree obtained from analysis of 76 *cytb* gene sequences from specimens of 18 species of *Phyllotis*. Numbers denote bootstrap support values for the adjacent nodes; only values for species clades and relationships among them are shown. *Inset* shows details of the clades of *P. x. rupestris* and *P. limatus*. Branch tips are labeled with GenBank accession number and, when available, museum catalog number. Labels for specimens from Volcán Llullaillaco are shown in color, and the altitudes of collection localities are indicated.

Phylogenetic analysis of *cytochrome b* (*cytb*) sequences corroborated the species identifications of our record specimens of *P. limatus* and *P. xanthopygus* and revealed close relationships with conspecific specimens from elsewhere in northern Chile, northern Argentina, and southern Peru (Fig. 2). The summit specimen (GD 2097) groups with those of previously collected altiplano specimens of *P. xanthopygus rupestris* (13). Moreover, the *cytb* haplotype of this summit specimen is identical to that of another *P. x. rupestris* specimen (LCM1780) collected at Toconao, Chile, a 2,500-m locality *ca*. 180 km NNE of Volcán Llullaillaco. Similarly, two other specimens of *P. x. rupestris* collected at different altitudes (GD 2082 at 4,406 m and GD 2095 at 5,069 m) on Volcán Lullaillaco share identical *cytb* haplotypes with a specimen (LCM1737) collected at the mouth of the Loa River on the Pacific coast, *ca*. 400 km NW of Volcán Lullaillaco. Thus, not only does *P. x. rupestris* range from sea level to the crest of the Andean Cordillera at 6,739 m (the broadest altitudinal distribution of any mammal), but individuals found at opposite extremes of this vast range share identical *cytb* haplotypes.

Our capture of *P. x. rupestris* on the summit of Llullaillaco suggests that we may have generally underestimated the altitudinal range limits and physiological tolerances of small mammals simply because the world’s high summits remain relatively unexplored by biologists. The upper range limits of many vertebrate taxa are not precisely demarcated, and putative altitudinal records for many taxa exist as unverified sightings or reports in mountaineering expedition accounts rather than as voucher specimens in museum collections.

Our discoveries prompt many evolutionary and ecological questions. Given the exceptionally broad altitudinal range of *P. xanthopygus*, have mice from the high Andes evolved genetically based adaptations to hypoxia that distinguish them from lowland conspecifics? To what extent is the ability to tolerate such a broad range of environmental conditions attributable to acclimatization (physiological plasticity)? Given that mice inhabiting the upper reaches of Llullaillaco are living >2,000 m above the upper limits of green plants, what are they eating? Such questions can be answered by future mountaineering expeditions in the Humboldtian tradition that combine high-altitude exploration and scientific discovery.

## Materials and Methods

### Specimen Collection.

We captured mice using Sherman live traps, except for the specimen from the Llullaillaco summit, which was captured by hand (Movie S2). We killed mice in the field and prepared them as museum specimens, all of which are housed at the Colección de Mamíferos of the Universidad Austral de Chile, Valdivia, Chile. Tissue samples from Argentinian and Peruvian specimens were obtained as loans from the collections of Centro Nacional Patagónico, Puerto Madryn, Argentina, and Louisiana State University Museum of Natural Science, Baton Rouge, LA.

All mice were collected in accordance with permissions to J.F.S. from the following Chilean government agencies: Servicio Agrícola y Ganadero (Resolución extenta #209/2020), Corporación Nacional Forestal (Autorización nos. 171219 and 1501221), and Dirección Nacional de Fronteras y Límites del Estado (Autorización de Expedición Cientifica #68). All mice were live-trapped and handled in accordance with protocols approved by the Institutional Animal Care and Use Committee at the University of Nebraska (Project ID 1919). Argentinian samples were exported under Permit #3938/03 from the Dirección de Fauna y Flora Silvestres.

### DNA Sequencing.

We sequenced the first 801 base pairs of the mitochondrial gene *cytb* from *Phyllotis* specimens collected from Volcán Llullaillaco and adjacent regions of Chile and Argentina.

### Phylogenetic Analysis.

We integrated newly generated sequences into a dataset containing GenBank sequences from *P. xanthopygus*, *Phyllotis bonariensis*, *Phyllotis caprinus*, and *P. limatus*. After excluding redundant sequences, the final matrix consisted of 76 sequences from 18 recognized species of *Phyllotis*. We used *Auslicomys pictus* and *Loxodontomys micropus* as outgroups. Using the HKY+I+G substitution model, we estimated the Maximum Likelihood *cytb* phylogeny with IQ-TREE (14); perturbation strength = 0.5, and the number of unsuccessful iterations = 100. Branch support was estimated via 1,000 bootstrap replicates.

## Supplementary Material

Supplementary File

Supplementary File

Supplementary File

## Data Availability

All DNA sequences are available in GenBank (MT183676 to MT183689).
