# Supplementary material for: Discovery of the world’s highest-dwelling mammal
Source: Proc Natl Acad Sci U S A. 2020 Jul 16;117(31):18169–71. doi: 10.1073/pnas.2005265117 (PMC7414144; doi:10.1073/pnas.2005265117)
Supplement: Supplementary File [file pnas.2005265117.sapp.pdf]

SI Appendix

Discovery of the world's highest-dwelling mammal

Jay F. Storz, Marcial Quiroga-Carmona, Juan C. Opazo, Thomas Bowen, Matthew Farson, Scott J. Steppan, and Guillermo D'Elía

Legends for Supplemental Movies

Movie S1. Footage of a leaf-eared mouse (*Phyllotis* spp.) at 6205 m on Volcán Llullaillaco, Región de Antofagasta, Chile (24°43.052'S, 68°33.323'W). Filmed by Matthew Farson.

Movie S2. Capture of a yellow-rumped leaf-eared mouse, *Phyllotis xanthopygus*, on the summit of Volcán Llullaillaco (6739 m), Región de Antofagasta, Chile (24°43.235'S, 68°32.208'W). Filmed by Mario Pérez Mamani.

www.pnas.org/cgi/doi/10.1073/pnas.2005265117
